# Feeding difficulties, a key feature of the *Drosophila* NDUFS4 mitochondrial disease model

**DOI:** 10.1242/dmm.032482

**Published:** 2018-03-01

**Authors:** Sarah Foriel, Julien Beyrath, Ilse Eidhof, Richard J. Rodenburg, Annette Schenck, Jan A. M. Smeitink

**Affiliations:** 1 Khondrion BV, Philips van Leydenlaan 15, 6525 EX, Nijmegen, The Netherlands; 2 Radboud Center for Mitochondrial Medicine (RCMM) at the Department of Pediatrics, Radboud University Medical Center, Geert Grooteplein Zuid 10, 6500 HB, Nijmegen, The Netherlands; 3 Department of Human Genetics, Donders Institute for Brain, Cognition and Behaviour, Radboud University Medical Center, Geert Grooteplein 10, 6525 GA, Nijmegen, The Netherlands

**Keywords:** Disease model, *Drosophila* model, Feeding impairment, Mitochondrial disease

## Abstract

Mitochondrial diseases are associated with a wide variety of clinical symptoms and variable degrees of severity. Patients with such diseases generally have a poor prognosis and often an early fatal disease outcome. With an incidence of 1 in 5000 live births and no curative treatments available, relevant animal models to evaluate new therapeutic regimes for mitochondrial diseases are urgently needed. By knocking down ND-18, the unique *Drosophila* ortholog of NDUFS4, an accessory subunit of the NADH:ubiquinone oxidoreductase (Complex I), we developed and characterized several dNDUFS4 models that recapitulate key features of mitochondrial disease. Like in humans, the dNDUFS4 KD flies display severe feeding difficulties, an aspect of mitochondrial disorders that has so far been largely ignored in animal models. The impact of this finding, and an approach to overcome it, will be discussed in the context of interpreting disease model characterization and intervention studies.

This article has an associated First Person interview with the first author of the paper.

## Abstract (precis)

**Summary:** The characterization of a novel *Drosophila* model of NDUFS4 mitochondrial disease revealed a pronounced defect in feeding abilities and recapitulated additional human disease features.

## INTRODUCTION

Among the five complexes forming the mitochondrial oxidative phosphorylation system (OxPhos), NADH:ubiquinone oxidoreductase, or Complex I (CI), is the largest and represents a privileged target for mutation. So far, mutations in 33 structural subunit genes are associated with human disease (Mayr et al., 2015). Forty-two of the 44 human CI subunits are conserved in *Drosophila melanogaster* (Garcia et al., 2017). NADH dehydrogenase (ubiquinone) Fe-S protein 4, 18 kDa (NDUFS4) is an accessory subunit of CI which stabilizes the assembly of the N-module to the Q-module (Guerrero-Castillo et al., 2017; Sánchez-Caballero et al., 2016). NDUFS4 inactivation leads almost invariably to Leigh syndrome and neurological symptoms (Koene et al., 2012). *NDUFS4* has a unique *Drosophila* ortholog called *ND-18* (herein referred to as dNDUFS4).

Targeting *Drosophila* OxPhos genes with RNA interference (RNAi) in a spatially and temporally controlled manner has previously contributed to the deciphering of mitochondrial pathophysiology (Copeland et al., 2009; Foriel et al., 2015; Sánchez-Martínez et al., 2006). Taking advantage of the *Drosophila* genetic tools available, we inactivated dNDUFS4 ubiquitously and in specific tissues. The need for relevant animal models that recapitulate the symptoms of a disease is crucial to understand and elucidate the pathomechanisms, and to later use the models to develop therapeutic strategies. Feeding difficulties have frequently been reported in patients with CI deficiencies (van den Engel-Hoek et al., 2017; Kisler et al., 2010; Koene et al., 2012, 2013; de Laat et al., 2015; Morava et al., 2006; Ramakers et al., 2017; Read et al., 2012; Smeitink, 2003), but have largely been neglected in animal models for mitochondrial disease. Here, we present a novel *Drosophila* model of CI deficiency which develops a dramatic feeding impairment, along with other symptoms recapitulating CI-deficient NDUFS4-mutated patient characteristics, such as a severely reduced lifespan, locomotor defects and signs of neurodegeneration.

## RESULTS

### Generation and validation of a NDUFS4 *Drosophila* model

Taking advantage of the UAS-Gal4 system and available resources (Dietzl et al., 2007; Flockhart et al., 2006), we induced ubiquitous knockdown of dNDUFS4, the *Drosophila* ortholog of human NDUFS4 (referred to as dNDUFS4 KD in this article). The efficiency of the dNDUFS4 knockdown in the RNAi flies was assessed by quantitative reverse transcription PCR (qRT-PCR). The ubiquitous dNDUFS4 KD flies showed only 20% of dNDUFS4 transcript, validating knockdown (KD) at the mRNA level (Fig. S1A). We further ruled out a potential integration of the utilized RNAi construct into the genomic landing site at cytogenetic position 40D (Fig. S1B), which was reported to be associated with off-target effects (Green et al., 2014; Manning et al., 2016).

**Fig. 1.**
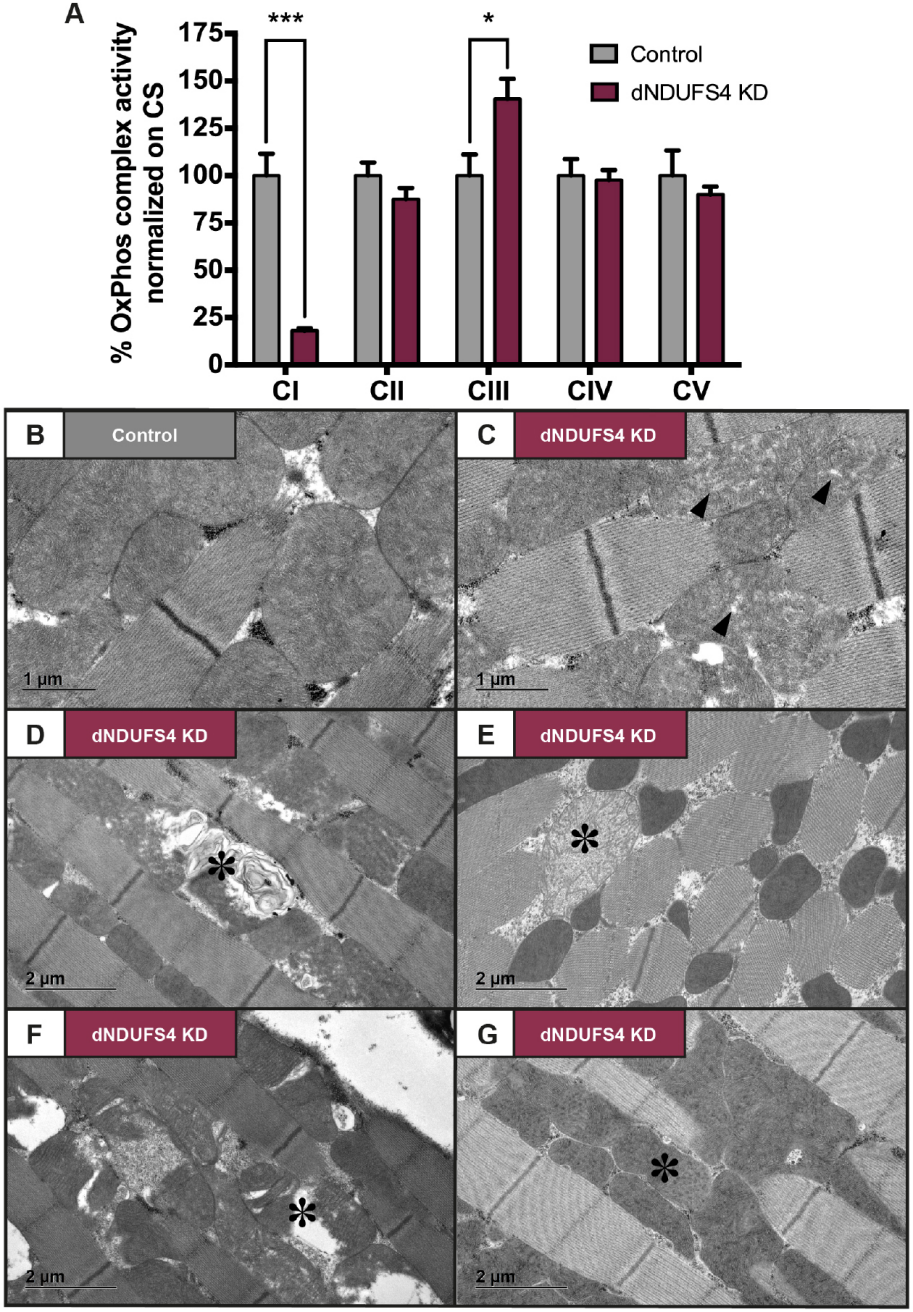
**Morphofunctional characterization of mitochondria in ubiquitous dNDUFS4 KD flies.** (A) Enzymatic activity measurement of the mitochondrial OxPhos complexes normalized on citrate synthase (CS). CI activity was reduced to 18.1%±2.8% (***P<0.0001) and CIII activity was increased to 140.4%±23.7% (*P<0.0102) in ubiquitous KD flies (mean± s.e.m., *n*=5, two-way ANOVA). (B-G) Transmission electron microscopy of indirect flight muscle. (B) Representative image of mitochondria in controls. (C) Representative image of mitochondria in dNDUFS4 KD flies. Mitochondria with dispersed cristae are indicated by black arrowheads. In D-G, asterisks indicate examples of mitochondria with structural defects found in ubiquitous KD fly samples. (D) Mitochondrion with onion-like inner membrane. (E) Swollen mitochondrion. (F) Mitochondrion with sparse matrix/distant cristae. (G) Mitochondria with irregular cristae densities. Scale bars: 1 µm in B and C; 2 µm in D-G.

We next determined the degree to which dNDUFS4 KD affects the enzymatic activity of CI and the other OxPhos complexes [Complexes II to V (CII-CV)]. The spectrophotometer measurements revealed a CI residual activity of 18% in the dNDUFS4 KD flies, with no significant effects on OxPhos CII, CIV and CV (Fig. 1A). CIII activity was increased in the ubiquitous KD flies, potentially representing a compensatory mechanism. These results show that ubiquitous dNDUFS4 KD led to isolated CI deficiency (Fassone et al., 2011; Swalwell et al., 2011).

The effects of dNDUFS4 KD on mitochondrial morphology were assessed by electron microscopy in the indirect flight muscle, a tissue requiring extensive energy production. Mitochondria in control flight muscles appeared intact and were characterized by compact lamellar cristae. Mitochondria in the flight muscle of dNDUFS4 KD flies presented with a wide range of structural abnormalities (Fig. 1B-G). Morphological defects consisted of dispersed cristae (Fig. 1C), onion-like swirling membranes (Fig. 1D), swollen mitochondria (Fig. 1E), sparse matrix (Fig. 1F) and irregular cristae densities (Fig. 1G).

Together, the reduced amount of dNDUFS4 transcripts and mitochondrial morphofunctional defects confirmed the dNDUFS4 KD flies as a model for CI deficiency.

### Ubiquitous dNDUFS4 KD flies display a severe feeding impairment

**Fig. 2.**
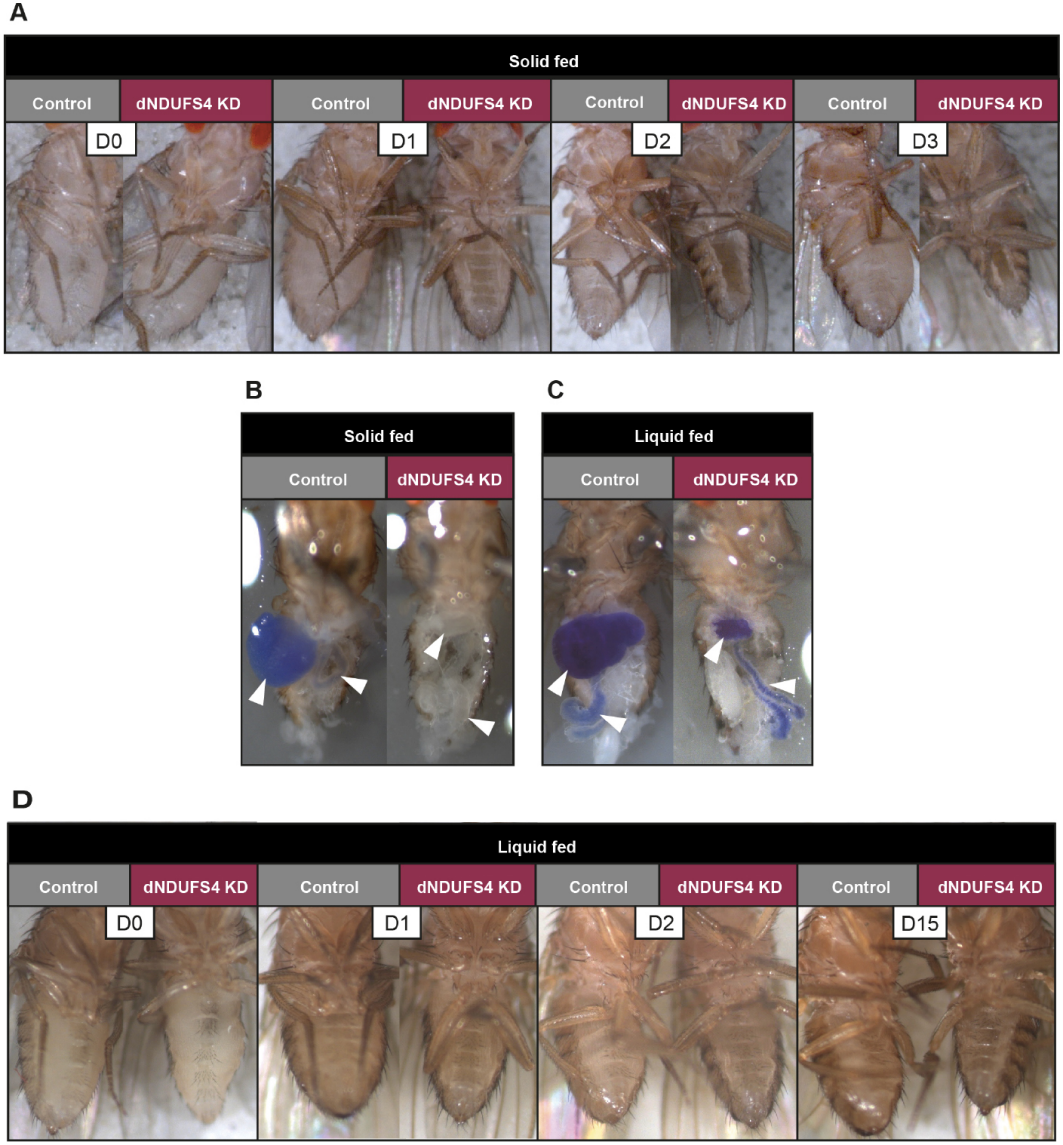
**The ubiquitous dNDUFS4 KD model presents feeding deficits.** Control flies are presented on the left and ubiquitous KD flies on the right, as indicated. (A) Representative images of abdomen evolution over time upon regular cornmeal feeding, highlighting a progressive shrinkage of the KD fly abdomen. All dNDUFS4 KD flies presented abdominal shrinkage. D0 represents the day of collection (0 to 1 day old), D1 represents 1-2 days old, etc. (B,C) Analysis of the digestive system upon solid and liquid food supplemented with 0.5% Bromophenol Blue. Upon regular cornmeal fly food, the digestive system of all ubiquitous KD flies appeared blue-free (B). When subjected to 5% sucrose with Bromophenol Blue delivered in capillaries, both control and ubiquitous dNDUFS4 KD flies were able to ingest liquid food formulation, although the KD flies seemed to eat qualitatively less (C). White arrowheads indicate the crop and gut. (D) Effects of liquid feeding on progressive abdomen shrinkage. The KD flies with liquid feeding presented a dramatically slower shrinkage compared with the solid-feeding flies (note the different last timepoint of the time series).

*Drosophila* pupae eclosion is a high energy-demanding process (Merkey et al., 2011). As a consequence, defects in oxidative phosphorylation can lead to eclosion defects. Quantification of eclosed and dead pupae 7 days after onset of eclosion in the culture vials revealed a significantly reduced eclosion rate (37% pupal eclosion) in ubiquitous dNDUFS4 KD flies compared with that (97%) in controls (*P*<0.0001) (Fig. S1C). A fraction of the eclosed dNDUFS4 KD adults appeared weak, fell onto the food and died rapidly. To ensure that falling onto the food was not causing the premature death, we kept vials horizontally. This did not improve the survival of the dNDUFS4 KD flies. Upon daily inspection, we observed that the surviving ubiquitous dNDUFS4 KD flies presented a dramatic and progressive shrinkage of the abdomen in the first days after eclosion (Fig. 2A) but laid eggs. To investigate whether this observation was caused by feeding defects, we exposed ubiquitous dNDUFS4 KD and control flies at larval and adult stages to regular cornmeal fly food supplemented with Bromophenol Blue. Upon intake, this dye colors the digestive system of the flies (crop, midgut and hindgut) and gives a qualitative indication of the food intake without apparent effect on fly development or health. The digestive system of third instar control larvae (Fig. S2A) and adult flies (Fig. 2B) displayed a strong blue color after 1 day on the dyed food. The midgut and hindgut of dNDUFS4 KD larvae showed less intense staining compared with the controls (Fig. S2A). No staining could be detected in the midgut and hindgut of adult ubiquitous KD flies, highlighting a striking feeding defect (Fig. 2B). Despite actively extending their proboscis to get in contact with the food, the ubiquitous KD flies failed to uptake food.

As the dNDUFS4 KD flies appeared unable to efficiently take up the regular agar-cornmeal medium in this formulation, we provided them with a 5% sucrose solution (‘liquid feeding’, as opposed to the regular ‘solid feeding’) dyed with Bromophenol Blue. Under these conditions, the digestive systems of both the dNDUFS4 KD and control flies were strongly stained, confirming their abilities to take up liquid food (Fig. 2C). In addition, the abdomen shrinkage phenotype of the solid fed dNDUFS4 KD flies was strongly delayed in the liquid-fed KD flies (Fig. 2A versus D). Therefore, providing the ubiquitous dNDUFS4 KD flies with a 5% sucrose solution appeared to alleviate, to a certain extent, the feeding difficulties encountered with the regular cornmeal fly food.

### Ubiquitous KD flies have a dramatic lifespan defect

**Fig. 3.**
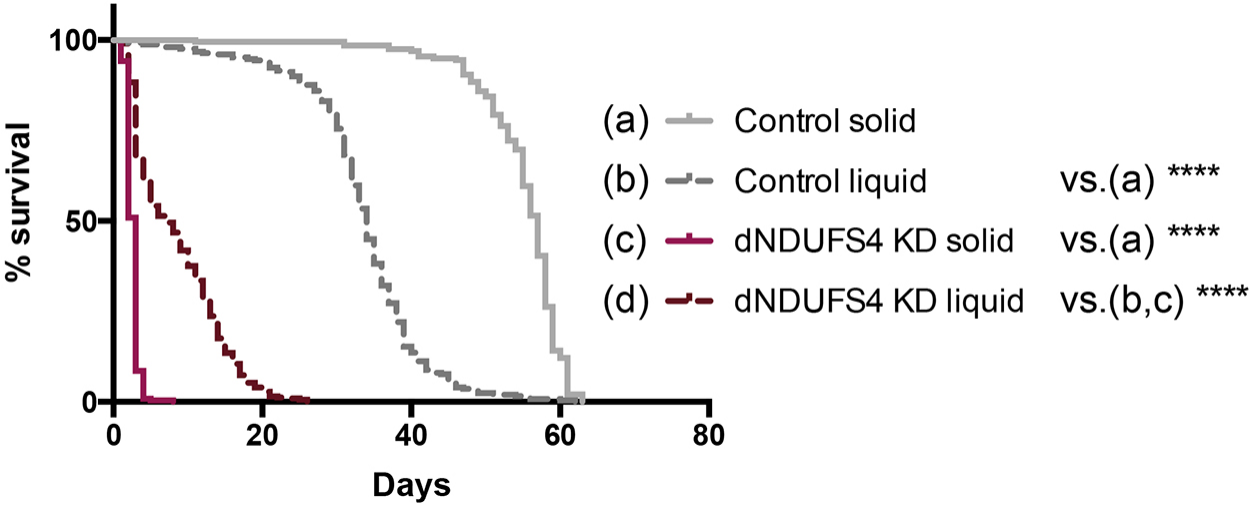
**Effects of solid and liquid feeding on the lifespan of control and ubiquitous dNDUFS4 KD flies.** Survival curve of ubiquitous dNDUFS4 KD flies and their corresponding controls fed with regular cornmeal medium or 5% sucrose. Solid-fed ubiquitous dNDUFS4 KD flies (*n*=198) die significantly faster than their genetic controls (*n*=222), with median survival (day at which 50% of the flies have died) at day 3 for KD flies and day 57 for control flies. When fed with 5% sucrose in capillaries, ubiquitous KD flies (*n*=325) displayed a reduced lifespan compared with control flies (*n*=249), with respective median survival at day 7 and day 34 (Kaplan–Meier curve, log-rank Mantel–Cox test, ****P<0.0001).

The outcome of patients with NDUFS4 mutations in general is poor, with death occurring in early childhood (Fassone and Rahman, 2012; Koene et al., 2012; Rahman et al., 1996). We therefore investigated the lifespan of the ubiquitous dNDUFS4 KD models. The surviving ubiquitous KD flies presented a dramatically reduced lifespan (*P*<0.0001) with a median survival (time at which 50% of the flies have died) of 3 days versus 57 days for the control flies (Fig. 3), consistent with the dramatically reduced lifespan of patients with NDUFS4 mutations. We next evaluated whether the feeding difficulty and resulting starvation were mainly responsible for the reduced lifespan observed in the dNDUFS4 KD flies. Indeed, 5% sucrose solution-fed dNDUFS4 KD flies survived significantly longer (*P*<0.0001) than their solid-fed siblings, with 7 days versus 3 days median survival, accounting only for a partial lifespan rescue (Fig. 3). It is worth noting that the control flies fed with 5% sucrose solution died significantly faster (*P*<0.0001) than the solid-fed control flies (Fig. 3). In addition, ubiquitous KD flies maintained on H_2_O only, had a similar median survival (of 3 days) to ubiquitous KD flies exposed to regular cornmeal food (Fig. S2B). These findings confirm that the ubiquitous KD flies have difficulties with ingesting nutrients, leading to a very short lifespan that could partially be rescued by liquid feeding with sucrose.

### Ubiquitous dNDUFS4 KD flies exhibit strong locomotion impairment

**Fig. 4.**
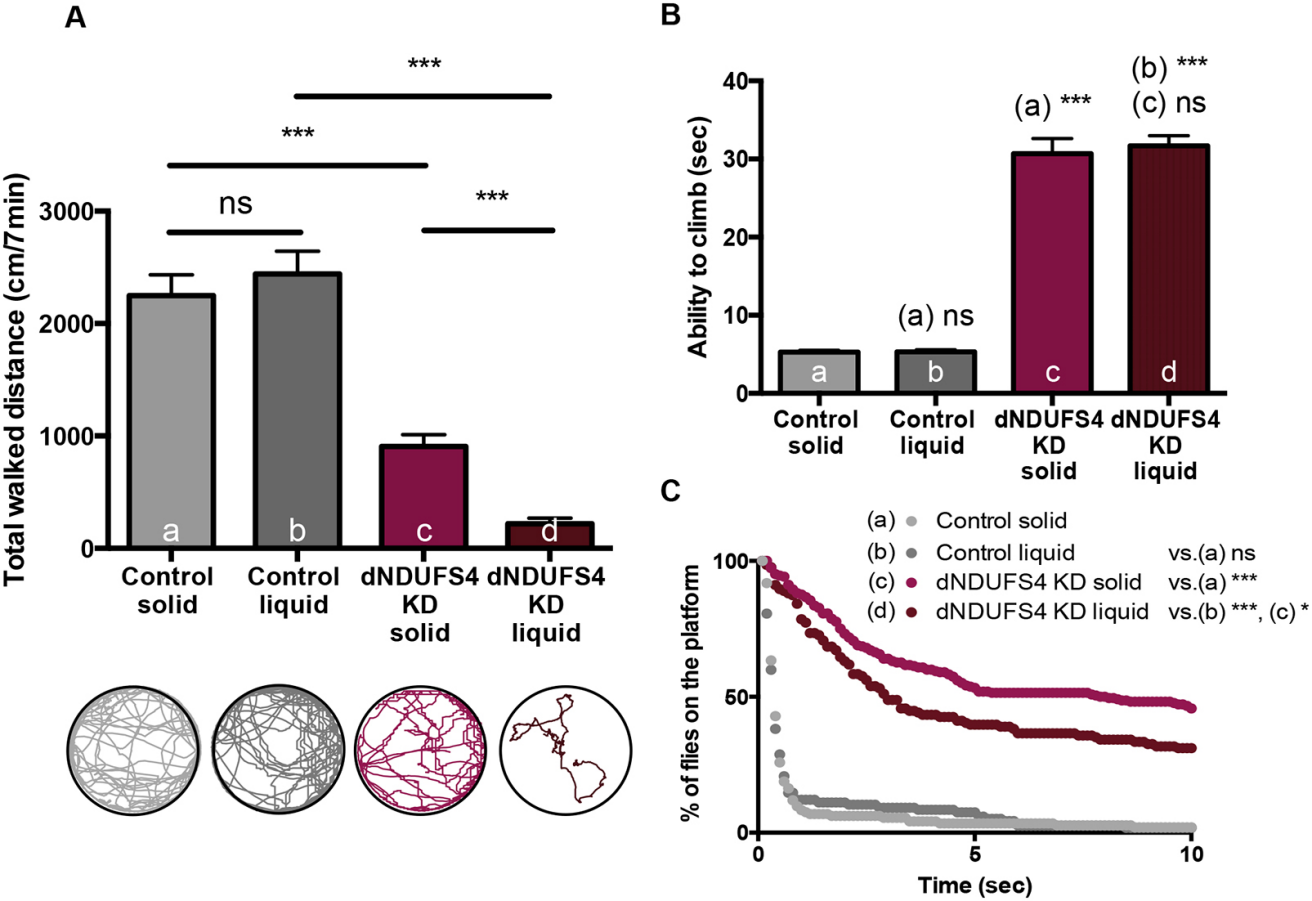
**Ubiquitous dNDUFS4 KD flies display locomotion impairments.** (A) The spontaneous locomotion assay highlighted a reduced total walked distance of solid-fed ubiquitous KD flies (905.6±105 mm/7 min; *n*=19) compared with controls (2248±185.3 mm/7 min; *n*=20). The liquid-fed dNDUFS4 KD flies had a severely reduced total walked distance over 7 min of acquisition (220.9±49.9 mm/7 min; *n*=18) compared with the controls (2441±202.7 mm/7 min; *n*=20) and their solid-fed siblings (***P<0.0001, unpaired Student's *t*-test, mean±s.e.m.; ns, nonsignificant). (B) Climbing abilities or time necessary for 70% of the flies to climb 9.5 cm. The climbing assay revealed impaired negative geotaxis of the solid-fed ubiquitous KD flies (30.7±1.920, *n*=8) compared with controls (5.2±0.2, *n*=4) (***P<0.0001). The liquid-fed ubiquitous dNDUFS4 KD flies also presented with negative geotaxis impairment (31.6±1.3 s, *n*=10) compared with the controls (5.3±0.2, *n*=4) (***P<0.0001, unpaired Student's *t*-test, mean±s.e.m.; ns, nonsignificant). Diet did not improve or impair the climbing abilities of ubiquitous KD flies (*P*<0.67). (C) Island assay. Solid-fed ubiquitous KD flies spent significantly (***P<0.0001) more time on the platform as expressed by the area under the curve (AUC) (685.2±156.7, *n*=8, for the controls; 6028±247.6, *n*=13, for the ubiquitous KD flies). The liquid-fed ubiquitous dNDUFS4 KD flies also spent significantly more time on the platform (AUC, 852.3±231.1; *n*=8) than the control flies (4727±584.2; *n*=8) (***P<0.0001). Liquid feeding improved the performances of ubiquitous KD flies in the island assay (*P<0.0293) (unpaired Student's *t*-test, mean±s.e.m.; ns, nonsignificant).

CI deficiency frequently results in loss of motor skills and fatigue, in addition to a plethora of other complaints. We set out to characterize locomotor abilities of ubiquitous dNDUFS4 KD and control flies by filming and tracking them when walking freely in an arena. The dNDUFS4 KD flies exhibited a significantly reduced walking distance compared with control flies (*P*<0.0001), with 905.6±105.0 mm versus 2248±185.3 mm per 7 min observation interval (Fig. 4A). They also showed a strong unbalanced gait and righting defects (Movie 1). Surprisingly, liquid feeding did not improve the locomotor abilities of ubiquitous dNDUFS4 KD flies. Liquid-fed KD flies walked significantly less (220.9±49.9 mm/7 min) than liquid-fed control flies (2441±202.7 mm/7 min, *P*<0.0001) or solid-fed dNDUFS4 KD flies (905.6±105.0 mm/7min, *P*<0.0001) (Fig. 4A). They also showed the characteristic unbalanced gait and righting impairment previously observed in solid-fed flies (Movie 1). These results are, however, in line with the fact that starvation induces locomotion activity in *Drosophila* (Connolly, 1966; Yang et al., 2015; Yu et al., 2016). Additionally, we did not observe differences in the average distance walked between solid- and liquid-fed control flies (*P*<0.4882) (Fig. 4A), suggesting that the nutrient limitation of the liquid feeding did not impair the locomotion of healthy flies. Because both the nutritional (fed or not) and health (CI deficient or not) status influence spontaneous locomotion of *Drosophila*, interpretation of the obtained data is challenging.

We therefore extended the characterization of locomotor abilities to include challenging conditions. The climbing assay relies on the natural behavior of flies to climb against gravity when tapped down, also referred to as negative geotaxis reflex. Compared with control flies, the ubiquitous dNDUFS4 KD flies displayed a significant climbing defect (*P*<0.0001). It took 5.2±0.2 s for 70% of the control flies to climb 9.5 cm, whereas the ubiquitous KD flies took 30.69±1.920 s (Fig. 4B). We did not observe a difference in climbing abilities between the solid- and liquid-fed dNDUFS4 KD flies (*P*<0.67), or between the solid- and liquid-fed control flies (*P*<0.9221) (Fig. 4B). We additionally assessed the challenged locomotor function in the island assay. In this test, flies were thrown onto a platform surrounded by water, triggering a flight escape response. The control flies left the platform within seconds, whereas dNDUFS4 KD flies were not able to efficiently initiate and/or execute the escape and remained substantially longer on the platform. This observation was quantified by averaging the area under the curve (AUC), and the control flies had a much smaller AUC (685.2±156.7) than the ubiquitous KD flies (6028±247.6; *P*<0.0001) (Fig. 4C). Interestingly, liquid feeding improved the flight escape abilities of dNDUFS4 KD flies when thrown on the platform in the island assay (*P*<0.0293), but had no significant effect on the flight escape abilities of the controls (*P*<0.5591) (Fig. 4C).

In conclusion, we show a partial amelioration in island assay performance in liquid-fed ubiquitous KD flies. The climbing abilities of these flies did not improve upon liquid feeding.

### dNDUFS4 KD flies display brain histological defects

**Fig. 5.**
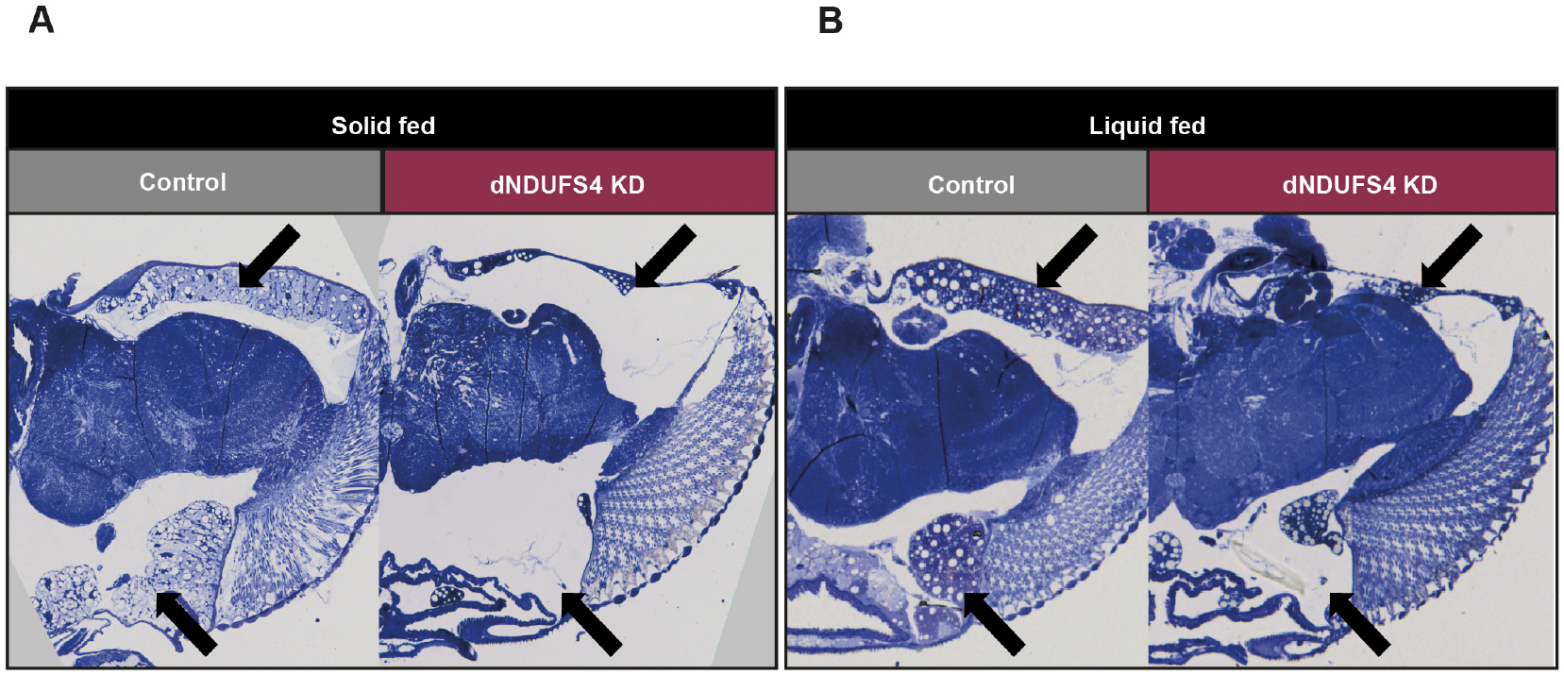
**Ubiquitous dNDUFS4 KD flies have a strongly reduced pericerebral fat body.** (A,B) Transverse sections of 1-day-old fly heads stained with Toluidine Blue. Black arrows point at the pericerebral fat body. The intensity of the histological staining, Toluidine Blue, is not indicative of differences in fat composition. (A) Control and ubiquitous KD flies fed with solid food. (B) Control and ubiquitous KD flies fed with liquid food. Both solid- and liquid-fed KD flies displayed dramatic pericerebral fat body depletion.

To further characterize the consequences of the feeding difficulties of adult flies, we analyzed lipid storage in head histological sections of solid- and liquid-fed animals. The solid-fed dNDUFS4 KD flies presented a drastic pericerebral fat body deficit compared with control flies of the same age and identical diet (Fig. 5A). This fat storage impairment is consistent with observations in other animal models of mitochondrial disease, but is poorly characterized and understood (Johnson et al., 2013; Lindfors et al., 2011; Tyynismaa et al., 2010; Wang et al., 2016). Interestingly, while we showed that successful feeding with 5% sucrose solution could improve the lifespan of the dNDUFS4 flies (Fig. 3), it had no detectable effect on the head fat body deficit (Fig. 5B). Together, these data emphasize the importance of feeding defects in CI deficiencies and suggest a contribution to a number of key phenotypes associated with CI disorders.

The histology sections also revealed neurodegenerative lesions in the central nervous system (CNS) of both the solid- and liquid-fed dNDUFS4 KD flies (Fig. 5A,B). Of note, we observed fewer lesions in the head sections of the liquid-fed dNDUFS4 KD flies compared with the solid-fed KD flies. This neurodegeneration is in line with the observed severe unbalanced gait and righting defects in both solid- and liquid-fed dNDUFS4 KD flies (Movie 1).

### Tissue-specific dNDUFS4 KD recapitulates the reduced lifespan and, partially, the locomotor defects of ubiquitous dNDUFS4 KD

**Fig. 6.**
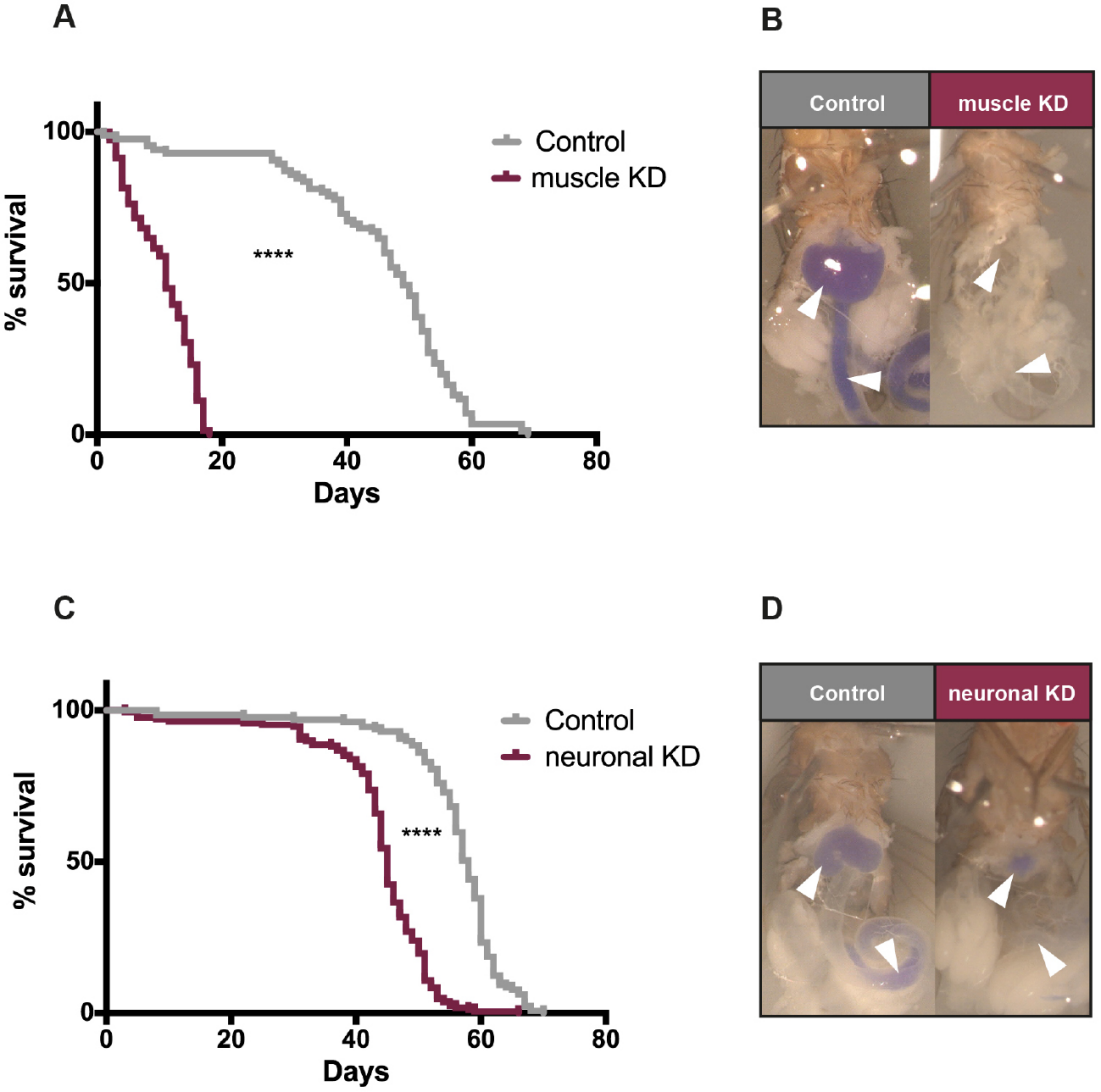
**Characterization of muscle- and neuron-specific NDUFS4 KD.** (A) Survival curve of flies collected at D0 fed with regular cornmeal fly medium. Muscle KD flies (*n*=151) die significantly faster than their genetic controls (*n*=85) (****P<0.0001), with a median survival of 11 and 49 days, respectively. (B) Abdomen photography of flies fed with regular cornmeal medium supplemented with 0.5% Bromophenol Blue highlighted important feeding difficulties, although the intestines showed a faint blue coloration. (C) The neuronal KD flies (*n*=167, median survival 45 days) died at an earlier age than their isogenic controls (*n*=129, median survival 58 days) (****P<0.0001) (Kaplan–Meier curve, log-rank Mantel–Cox test, GraphPad Prism 6). (D) Food supplemented with 0.5% Bromophenol Blue also highlighted feeding difficulties, but with a milder degree of severity. White arrowheads indicate the crop and gut.

Mitochondrial diseases are phenotypically highly heterogeneous multisystemic disorders. To elucidate the potential contribution of muscles (e.g. in the proboscis or intestine) to the feeding phenotype, we selectively knocked down dNDUFS4 in differentiated muscle using the Mhc-Gal4 driver. The maximal lifespan of the muscle KD flies was significantly reduced (*P*<0.0001), with a median survival of 11 days for the KD flies compared with 49 days for the control flies (Fig. 6A). Importantly, the muscle KD faithfully reproduced the feeding difficulties, as depicted by only very faint blue staining of the digestive system (Fig. 6B). It also reproduced the locomotor impairments of the ubiquitous KD, as illustrated by an unbalanced walk and righting defect (Movie 1). Thus, the muscle KD recapitulated the lifespan, feeding and locomotor defects of the ubiquitous dNDUFS4 KD flies.

Because neurological involvement is often prominent in mitochondrial disease, we also selectively knocked down dNDUFS4 in the brain using the panneuronal elav-Gal4 driver. Once more, the lifespan of the neuronal KD flies was significantly affected (*P*<0.0001), with a median survival of 45 days for the neuronal KD flies versus 58 days for the control flies (Fig. 6C). The neuronal dNDUFS4 KD flies exhibited a mild decrease in food uptake in comparison to the control flies (Fig. 6D). On the other hand, the neuronal dNDUFS4 KD flies did not present locomotor defects (neither gait unbalance nor righting defect), but did exhibit a progressive impairment in the negative geotaxis response, a crucial innate behavior in flies, in both solid and liquid feeding conditions (Movie 2).

These findings highlight the broad spectrum of clinical presentations and organ involvement recapitulated by the dNDUFS4 KD *Drosophila* models, and indicate that the overall phenotypes of ubiquitous dNDUFS4 KD originate from multi-organ defects.

## DISCUSSION

We here report new ubiquitous and tissue-specific knockdown models for mitochondrial CI deficiency, induced by targeted knockdown of the *Drosophila* NDUFS4 ortholog, dNDUFS4. The relevance of a *Drosophila* model for mitochondrial disease lies in its capacity to recapitulate the fundamental characteristics of the disease (Foriel et al., 2015; Pandey and Nichols, 2011; Sen and Cox, 2017). The dNDUFS4 model reported here shows key features of mitochondrial disease with a clear isolated CI deficiency (Fig. 1A) and structure anomalies of mitochondria (Fig. 1B-G). Like patients with Leigh syndrome, *Ndufs4^−/−^* mice (Kruse et al., 2008) and some other Leigh-like *Drosophila* models (Da-Rè et al., 2014), the dNDUFS4 KD flies display a dramatic lifespan reduction (Fig. 3) as well as histologic and behavioral signs of neurodegeneration (Fig. 5A,B; Movies 1 and 2). Impairment of patients' motor skills is well documented by the six-minute walking test or the GAITRite Walkway system (Ramakers et al., 2017; Stephenson et al., 2015), and has also been demonstrated in murine models using the rotarod, rope grip and CatWalk gait analyses (de Haas et al., 2016). The present study, thorough locomotor assessment with multiple assays, demonstrated a severe motor impairment of the dNDUFS4 KD flies as exemplified by an unbalanced walking pattern (Movie 1), reduced walking distance (Fig. 4A), and defects in climbing and flight responses (Fig. 4B,C). Further observations led to the identification of a dramatic feeding defect that could be partially improved by a liquid feeding approach with a 5% sucrose solution (Fig. 2A-D). In summary, the dNDUFS4 KD models recapitulate important features and characteristics of human CI disorders, making them relevant models with which to study mitochondrial disease.

Reduced food intake or malnutrition has dramatic consequences on the clinical outcome of patients with mitochondrial disease (Morava et al., 2006). Often, patients with nuclear-encoded CI deficiency and mitochondrial diseases are suffering from hypotonia, fatigue during mastication, feeding problems/starvation, swallowing difficulties, vomiting and dysphagia (van den Engel-Hoek et al., 2017; Koene et al., 2012, 2013; Morava et al., 2006; Noorda et al., 2007; Parikh et al., 2009; Wortmann et al., 2009). The feeding phenotype has been poorly studied in animal models of mitochondrial disease. In 2010, Tyynismaa et al. described in skeletal muscles of Deletor mice a transcriptional response mimicking amino acid, lipid starvation and induction of fasting-related hormone fibroblast growth factor 21 (Fgf21) (Tyynismaa et al., 2010). Deletor mice express a dominant patient mutation in the mitochondrial replicative helicase Twinkle that results in accumulation of mtDNA deletions and progressive respiratory chain deficiency. A second model was reported by Lindfors et al. in 2011, and makes the link between mitochondrial/CI dysfunction and anorexia in an *anx/anx* mouse model (Lindfors et al., 2011). In fact, the NADH dehydrogenase (ubiquinone) 1 alpha subcomplex, assembly factor 1 (*Ndufaf1*) gene was found to be located in the interval of the *anx* gene and was downregulated in the *anx/anx* mice. Aside from these two models, one can find mentioned in the literature food provided *ad libitum* to facilitate the nutrition of weak animals, such as *Ndufs4*^−/−^ mice (Alam et al., 2015; Choi et al., 2017; Karamanlidis et al., 2013; Kruse et al., 2008; Quintana et al., 2010, 2012).

*Drosophila* normally feed on microorganisms growing on the surface of fruits (nature) or gelatinous cornmeal medium (laboratory) by rhythmic, repetitive extensions and retractions of their proboscis (Itskov et al., 2014; Pool and Scott, 2014). dNDUFS4 KD flies display normal feeding behavioral modules (Pool and Scott, 2014) and try to feed on agar-cornmeal medium by extending and retracting their proboscis extensively, but the meal consumption appears to not be efficient with the consequence that eclosed flies rapidly die after pupal eclosion. Because sweet food stimulates consumption in *Drosophila* (Joseph et al., 2017), we provided 5% sucrose to the ubiquitous dNDUFS4 KD model in liquid form, expecting to trigger a feeding response. In the literature, liquid feeding refers to sucrose solutions of various concentrations (Diegelmann et al., 2017; Manzo et al., 2012; Qi et al., 2015; Ro et al., 2014). The 5% sucrose solution used in this work was clearly ingested and partially alleviated the effects of starvation on the lifespan and some locomotor aspects of CI-deficient flies. It would be interesting to further investigate the effects of different concentrations of sucrose or a more complete liquid diet (Deshpande et al., 2014; Gasque et al., 2013; Ja et al., 2007) to mimic the dietary intervention and optimization of nutritional intake applied in patients (Morava et al., 2006; Parikh et al., 2009). The administration of a more complete diet might, however, be challenging when delivered in capillaries because of the high viscosity of the solution and potential for increased thickening due to evaporation. Although pure sucrose solution is not an ideal medium in terms of nutrient constitution and will ultimately affect animal health, it offers greater options for experimental therapeutic dosing and uptake.

Spontaneous locomotor activity differs from challenged activity by the absence of external stimulus (Knoppien et al., 2000). Typically, the locomotion assay refers to spontaneous locomotion. In this assay, dNDUFS4 KD flies in the solid-feeding group which do not manage to feed walk significantly more, probably because they are foraging for food sources (Connolly, 1966; Knoppien et al., 2000; Yu et al., 2016). On the other hand, liquid-fed dNDUFS4 KD flies remain quiescent, probably to save energy, owing to their overall weakness resulting from the CI deficiency and lack of need to search for food. These results illustrate the complex interpretation of spontaneous locomotion as readout in models with feeding problems, because the distance walked by the diseased flies is also influenced by this phenotype (Martin, 2003). This, in turn, highlights the importance of identifying feeding difficulties in animal models of mitochondrial disease as this condition might interfere with the outcome and interpretation of the data. Interestingly, when comparing solid- and liquid-fed dNDUFS4 KD flies in the island assay (a challenged locomotion assay), we could observe an improvement of the flight escape upon a liquid diet. These results confirm the relevance of carefully choosing the readouts or assays to be examined according to the characteristics of the model to be used.

Among the various signs and symptoms studied in the dNDUFS4 KD model, only the pericerebral fat body deficit and climbing abilities were not improved by the liquid diet. Starvation in the solid-fed dNDUFS4 KD flies and nutrient restriction in the liquid-fed KD flies can possibly explain the mobilization of the lipid reserves and therefore reduced pericerebral fat body thickness to produce the energy to survive (Arrese and Soulages, 2010; Kühnlein, 2011; Rajan and Perrimon, 2013). Noteworthy, the pericerebral fat body in control flies was not affected by the 5% sucrose solution, suggesting that the fat body deficit observed in dNDUFS4 KD flies could originate independently from, or prior to, the feeding condition, and highlights a primary mitochondrial defect.

Also, the climbing response is not improved by liquid food administration and this specific parameter is strongly impaired in the neuronal dNDUFS4 KD flies, providing evidence for neurodegeneration. Both phenotypes might well reflect primary and severe mitochondrial defects. Yet, phenotypes that are ameliorated upon liquid feeding and therefore reversible by nature, such as lifespan, locomotion and island escape, could represent suitable outcomes to assess therapeutic efficacy.

The challenges of treating mitochondrial diseases lie in their extreme heterogeneous clinical presentations (Fassone and Rahman, 2012; Koene et al., 2012; Koenig, 2008; Rahman et al., 1996). With the dNDUFS4 KD fly model, we were able to establish a library of phenotypes recapitulating this heterogeneity. These can be further used for therapeutic evaluation (Foriel et al., 2015; Pandey and Nichols, 2011). The high level of complexity and severity might, however, impair the development of therapeutic approaches. Importantly, most mitochondrial diseases involve multiple organ systems and prominently neurological and myopathic features (Chinnery, 2014). As an alternative to ubiquitous KD, tissue-specific KD approaches could be used. In our study, both muscle and neuronal dNDUFS4 KD recapitulated specific phenotypes observed in ubiquitous KD, such as reduced lifespan, feeding difficulties, locomotor impairment and climbing defect. These tissue-specific KD models might, therefore, allow a more refined therapeutic approach by targeting specific phenotypes according to the drug of interest.

In summary, multiple lines of evidence establish the dNDUFS4 KD flies as new, highly disease-relevant models of CI deficiency. These models emphasize the importance of detecting feeding difficulties, and its existence in relation to the interpretation of results. It can finally be applied to study mitochondrial disease pathomechanisms and present several advantages for compound testing perspectives.

## MATERIALS AND METHODS

### *Drosophila* stocks and stock maintenance

The conditional RNAi line targeting mitochondrial CI subunit dNDUFS4 (vdrc101489), its genetic background (vdrc60100) and the UAS-Dicer-2 (vdrc60008) strains (Dietzl et al., 2007) were obtained from the Vienna *Drosophila* Resource Center (VDRC). A second RNAi line (vdrc42983) and its genetic background (vdrc60000) were also recruited and tested, but were found to be ineffective [neither induced KD nor the impaired feeding phenotype (Fig. S3A,B)]. Therefore, vdrc101489 was used for the RNAi line and vdrc60100 was used for the control. The actin-Gal4/TM6c, Sb Tb driver was obtained from Christiane Zweier, Humangenetisches Institut, Germany. The panneuronal elav-Gal4 (BL8760) and the muscle Mhc-Gal4 (BL55132) drivers were obtained from the Bloomington *Drosophila* Stock Center (Indiana University, USA). The driver w^1118^; UAS-Dicer-2; elav-Gal4 was assembled in house. All stocks were maintained on standard cornmeal-agar medium at 25°C in a 12 h:12 h light-dark cycle.

### Ubiquitous and tissue-specific dNDUFS4 KD

KD was achieved using the modulable binary UAS/Gal4 system (Brand and Perrimon, 1993). Ten virgin females from the UAS-RNAi line or its isogenic control line were crossed with ten Gal4 line males at 28°C and 60% humidity. The progeny of the crosses carrying both Gal4 driver and UAS-RNAi constructs are referred to as ubiquitous KD when the actin-Gal4 driver was used, muscle KD when the Mhc-Gal4 was used, neuronal KD when UAS-dicer2; elav-Gal4 was used, and as control flies when the genetic background of the dNDUFS4 UAS-RNAi line was crossed with either of the three respective drivers. After 24 h premating, crosses were transferred to the ‘experimental vial’ for 2-3 days to allow adequate population density. Fifteen days after the transfer on experimental vial, the total number of pupae, empty pupae and dead pupae were counted for each vial to evaluate the eclosion rate and pupae lethality. The day of fly collection is referred to as day 0 (D0) and corresponds to flies with an age of 0 to 1 day. Flies were either transferred to standard cornmeal-agar medium, referred to as ‘solid fed’, or to 5% w/v sucrose/Milli-Q H_2_O solution provided in haematocrit capillaries 75 mm/75 ul (Hirschmann Laborgeraete), referred to as ‘liquid fed’. For all the experiments, unless specified otherwise, female flies (D1, 1-2 days old) were used.

### qRT-PCR

Primers spanning the junction between exon 2 and 3 were designed with the software Primer 3 Plus (www.bioinformatics.nl/cgi-bin/primer3plus/primer3plus.cgi) using the *Drosophila melanogaster* dNDUFS4 (CG12203) nucleic sequence from the Ensembl database (http://www.ensembl.org/index.html). dNDUFS4 forward primer: 5′-AAGATCACCGTGCCGACTG-3′ and dNDUFS4 reverse primer: 5′-GACAATGGGTCGCCGCTG-3′ were used. Total RNA from control and ubiquitous KD flies were isolated using an RNeasy Lipid Tissue Mini Kit (Qiagen), according to the manufacturer's instructions, and treated with DNase I (Ambion). RNA concentration was measured with Qubit (Thermo Fisher Scientific) and cDNA synthesis was performed with a Bio-Rad iScript™ Reverse Transcription Supermix. qPCR was performed using SyBR Green (Promega). Relative dNDUFS4 gene expression was determined against the geometric mean of two housekeeping genes: γ-tubulin and polymerase 2. For each genotype, three biological replicates and two technical replicates were performed. Student’s *t*-test was performed using Prism 6 (GraphPad software, LaJolla, CA) to determine statistical significance (*P<0.05; **P<0.01; ***P<0.001).

### Validation of hairpin insertion locus

DNA of individual flies from the isogenic control line vdrc60100 (negative control, no hairpin), the *G9a* RNAi line vdrc110662 (positive control, double insertion at 30B and 40D) and the dNDUFS4 RNAi line vdrc101489 were isolated in DNA isolation buffer and proteinase K (Thermo Fisher Scientific). Diagnostic PCR was performed according to the guidelines provided by Green et al. (2014). Sequence 5′→3′: forward primer for the off-target associated 40D pKC43 site 5′-GCCCACTGTCAGCTCTCAAC-3′; forward primer for the 30B pKC43 integration site: 5′-GCTGGCGAACTGTCAATCAC-3′; reverse primer for pKC26: 5′-TGTAAAACGACGGCCAGT-3′. PCR using the 40D pKC43 forward primer in combination with the pKC26 reverse primer results in a ∼450 bp product in case of insertion; a ∼600 bp product is obtained in case of insertion when using the 30B PKC43 forward primer together with the pKC26 reverse primer.

### OxPhos enzymatic activity

Solid-fed 1-day-old (D1) flies were collected, snap frozen in liquid nitrogen in groups of 30 (five replicates), and preserved at −80°C. The mitochondrial fraction was isolated after homogenizing the flies with a glass pestle in 1mL SETH buffer (0.25 M sucrose, 2 mM EDTA-K, 10 mM Tris, 5×10^4^ U/l heparin) and centrifugation at 600 ***g*** for 10 min at 4°C. Two 200 µl aliquots of the 600 ***g*** supernatant were used for the OxPhos measurement (CI, CII, CII and CIV) following previously described methods (Rodenburg, 2011). The remaining samples were centrifuged at 14,000 ***g*** for 10 min at 4°C. The pellet was subsequently resolubilized in 400 µl Tris HCl (pH 7.6) for Complex V activity. The measurements were realized with a Konelab 20×Ti instrument (Thermo Fisher Scientific). Complex activities were normalized against citrate synthase activity and presented as percentages of the averaged values of the control flies. A multiple comparison test with Bonferroni correction was performed using GraphPad Prism 6 to determine the statistical significance of differences (*P<0.05; **P<0.01; ***P<0.001; ****P<0.0001).

### Histology and electron microscopy

Ubiquitous dNDUFS4 KD flies and their controls were collected (at D0) and fed with regular solid food or 5% sucrose for 1 day (D1). Heads and thoraces were bisected and fixed overnight in 2% glutaraldehyde buffered with 0.1 M sodium cacodylate, pH 7.4. Subsequently, the samples were postfixed for 1 h in 1% osmium teroxide in Palade buffer, pH 7.4, with 0.5% potassium hexacyanoferrate (III) trihydrate, dehydrated in ethanol and propylenoxide and embedded in Epon. Semithin, 0.5-1 µm transverse and longitudinal sections were stained with 1% Toluidine Blue. Ultrathin sections were stained with uranyl acetate and lead citrate and examined in a JEOL 1200 electron microscope.

### Lifespan

Newly eclosed flies (D0) were collected in groups of 20 per vial with a minimum of four replicates per genotype and maintained at 28°C. They were transferred to fresh cornmeal-agar medium every 2-3 days. The 5% sucrose solution (or Milli-Q H_2_O for starvation) was provided in capillaries that were refilled every day. Each experiment was repeated at least two times. Kaplan–Meier curves and statistical analysis was performed according to GraphPad Prism 6 survival function, with a log-rank Mantel–Cox test to determine statistical significance (*P<0.05; **P<0.01; ***P<0.001; ****P<0.0001).

### *Drosophila* images

Adult flies of the same age (minimum *n*=5) were collected and maintained under CO_2_ anesthesia and/or dissected in 1× PBS for abdominal photography upon aging. Extended focus images were acquired using Axiovision software. The addition of Bromophenol Blue 0.5% (w/v) to the food (solid or liquid) allowed for qualitative evaluation of feeding abilities of larvae and adult flies (Amcheslavsky et al., 2014). Pictures were taken after 1 day feeding on food supplemented with Bromophenol Blue. All images show representative examples of the population.

### Locomotion, climbing and island assay

D0 flies were aliquoted in groups of five or 10 flies and allowed to recover from CO_2_ anesthesia for 24 h (on cornmeal agar or 5% sucrose fed). For locomotion tracking, five flies were placed in an arena of 3.7 cm diameter and 0.3 mm height. After 5 min adaptation, the video acquisition was started. Locomotion was recorded using a Logitec webcam and the HandyAvi program. The time lapse was set to 10 images/s, and 7 min were recorded. Tracking was performed with Ctrax and FixErrors Matlab GUI using the guidelines of Branson et al. (2009). For the climbing assay, 10 flies were placed in a vial of 23 mm diameter and 18.5 cm height, and allowed to acclimate for 5 min. The time required for seven flies (70% of the population) to cross a mark at 9.5 cm height after tapping them down was assessed over three trials in blinded experiments. Finally, the island assay was performed according to Eidhof et al. (2017).

## Supplementary Material

Supplementary information

First Person interview
