# Supplementary material for: Feeding difficulties, a key feature of the Drosophila NDUFS4 mitochondrial disease model
Source: Dis Model Mech. 2018 Mar 1;11(3):dmm032482. doi: 10.1242/dmm.032482 (PMC5897729; doi:10.1242/dmm.032482)
Supplement: Supplementary information [file dmm-11-032482-s1.pdf]

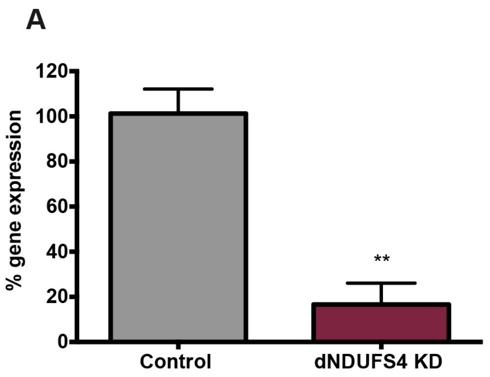

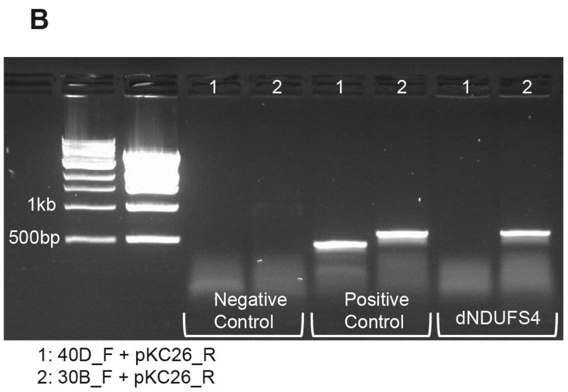

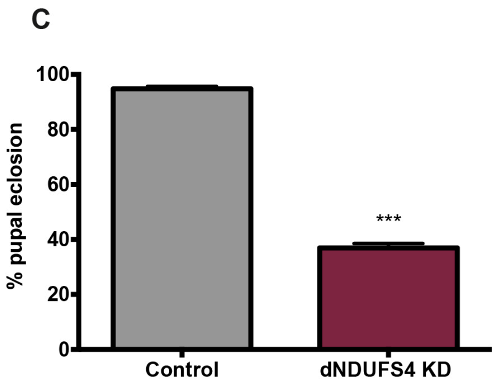

Figure S1. Validation of the NDUFS4 KD model
(A) Measurment of the transcript level in D1 ubiquitous KD flies (RNAi line vdrc101489) and their controls (vdrc60100). The qRT- PCR highlighted a reduction of 80% in the level of dNDUFS4 mRNA level in KD flies. Unless stated otherwise, the lines used in the manuscript refer to vdrc101489 for the RNAi line and vdrc60100 for the control. **(B)** Diagnostic PCR according to Green et al., 2014. Vdrc60100, the genetic background of vdrc 101489 (dNDUFS4 RNAi line) and negative control (without transgene insertion). Vdrc110662 (G9a RNAi line) is the positive control and presents a transgene insertion in both the 40D and 30B positions. The diagnostic PCR validated the transgene insertion in the 30B site in the vdrc101489 KK line. **(C)** The eclosion rate corresponds to the number of eclosed pupae over the total number of pupae. The ubiquitous KD flies display an eclosion rate of

Disease Models & Mechanisms • Supplementary information

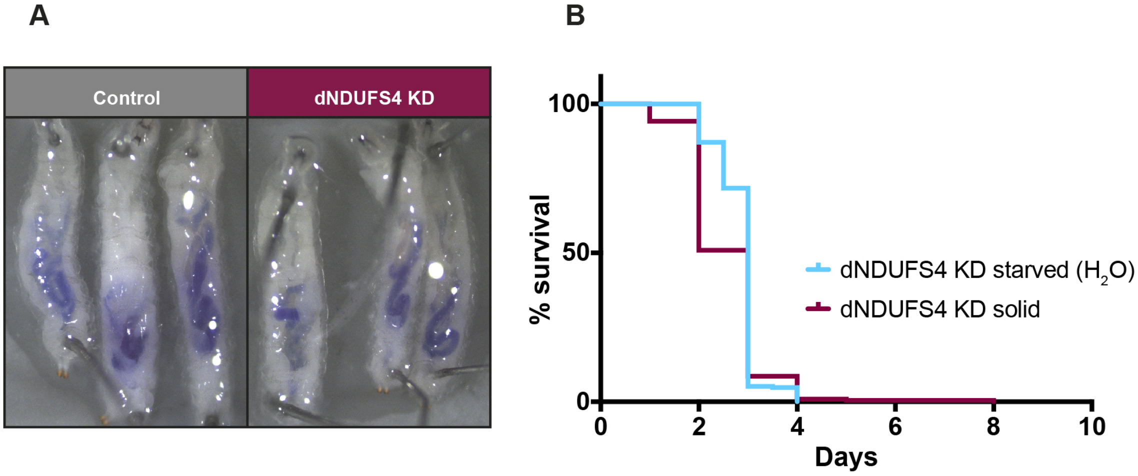

Figure S2. Characterization of feeding
(A) Pictures of larvae fed during one days with cornmeal food supplemented with bromophenol blue. Both control and ubiquitous KD larvaes are able to feed on solid food although blue staining in the KD larvae is diminished. **(B)** Comparison of survival curve of the ubiquitous KD flies (n=222) upon solid food and starvation (H2O only, n=272). The solid fed KD flies live significantly longer than the starved flies p<0.0002, although they both present a median survival of 3 days

Disease Models & Mechanisms • Supplementary information

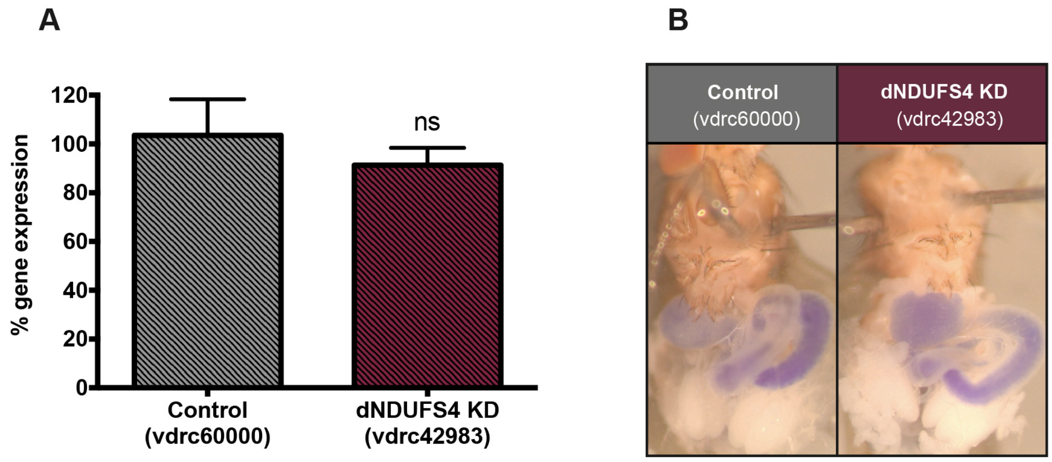

Figure S3. Characterization of a second RNAi line

(A) Measurment of the transcript level in D1 ubiquitous KD flies and their controls. With the RNAi line vdrc42983 and its control vdrc 60000, the qRT- PCR instead showed no reduction in the level of dNDUFS4 mRNA level in KD flies. **(B)** Pictures of D1 adult flies (vdrc42983 and vdrc60000) fed during one days with cornmeal food supplemented with bromophenol blue. Both control and KD present blue crop, midgut and hindgut synonym of no obvious feeding impairment. Unless stated otherwise, the lines used in the manuscript refer to vdrc101489 for the RNAi line and vdrc60100 for the control.

Supplemental Movies

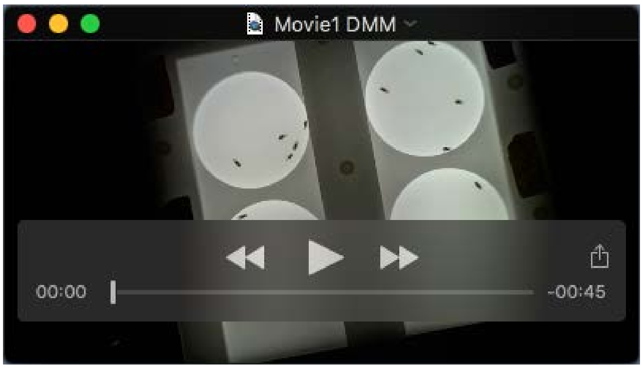

Movie 1: Video of the locomotion assay displaying ubiquitous KD flies and their control upon solid and liquid food at D1. Upper left arena: KD solid fed, upper right arena: KD liquid fed, lower left arena: Control solid fed, and lower right arena: control liquid fed. The ubiquitous KD flies present righting defects and unbalance gait.

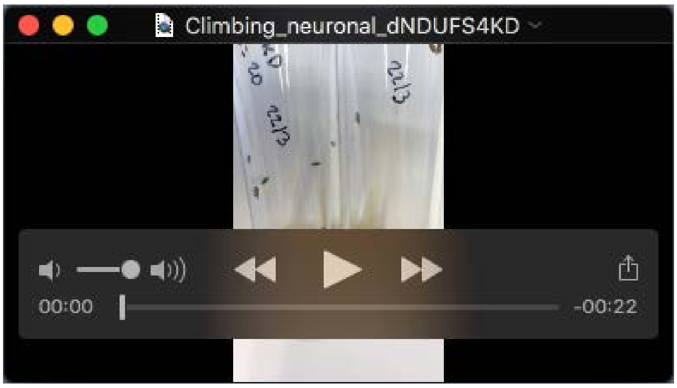

Movie 2: Video example of the negative geotaxis impairment in D25 neuronal KD flies (left) compared to their control (right).

Disease Models & Mechanisms • Supplementary information
