# Supplementary material for: Feeding difficulties, a key feature of the Drosophila NDUFS4 mitochondrial disease model
Source: Dis Model Mech. 2018 Mar 1;11(3):dmm032482. doi: 10.1242/dmm.032482 (PMC5897729; doi:10.1242/dmm.032482)
Supplement: First Person interview [file dmm-11-032482-s2.pdf]

FIRST PERSON

First person – Sarah Foriel

First Person is a series of interviews with the first authors of a selection of papers published in Disease Models & Mechanisms, helping early-career researchers promote themselves alongside their papers. Sarah Foriel is first author on 'Feeding difficulties, a key feature of the *Drosophila* NDUFS4 mitochondrial disease model', published in DMM. Sarah is a PhD student in the lab of Dr Jan Smeitink at Khondrion BV, Nijmegen, The Netherlands, investigating *Drosophila* as a model to study therapeutic approaches for mitochondrial diseases.

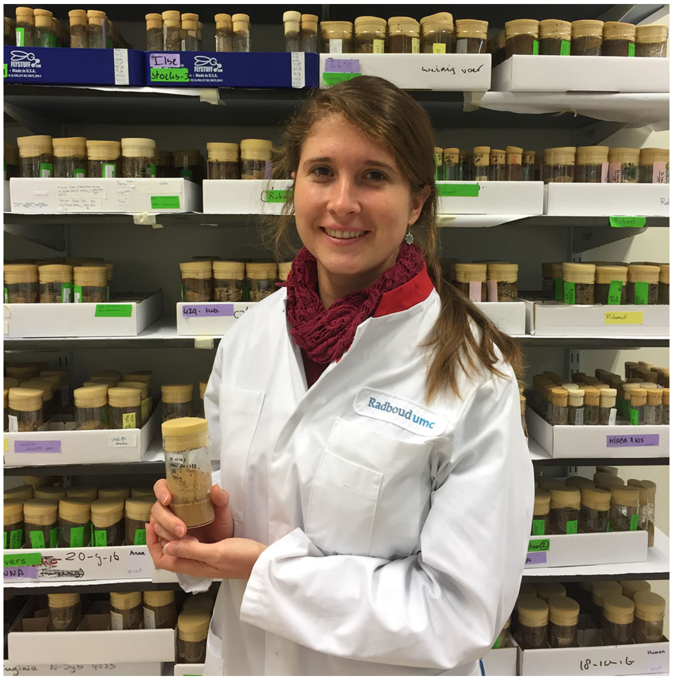

Sarah Foriel

How would you explain the main findings of your paper to non-scientific family and friends?

Mitochondria are the powerhouses of cells. When mitochondria are not working properly, the production of energy is threatened and energy-demanding organs such as the brain, muscles and many more suffer and start to fail, leading to devastating consequences for patients. With no treatments available, the need for relevant models to allow the development and evaluation of new therapeutics is urgent. By using the fruit fly to model mitochondrial deficiencies, we could recapitulate key features of the disease such as reduced lifespan, impaired locomotion and neurodegeneration. Additionally, patients with mitochondrial deficiencies often experience difficulties as they swallow, chew and eat, a characteristic poorly studied in animal models of mitochondrial disease. In the fruit fly, a feeding impairment compromises drug administration and testing, affects behaviours such as locomotion, and limits the choice of assays to use and how data can be interpreted. This impairment is therefore a crucial characteristic to be studied further.

What are the potential implications of these results for your field of research?

Feeding difficulties are largely underestimated in animal models of mitochondrial diseases. Besides the obvious challenge for drug administration and dosage, these feeding difficulties also interfere with the outcome of assays such as spontaneous locomotion in flies. Our findings therefore highlight the importance of carefully adapting the choice of assays to the model of interest, and the limitations on data interpretation.

What are the main advantages and drawbacks of the model system you have used as it relates to the disease you are investigating?

Drosophila melanogaster offers a large panel of possibilities in terms of genetics (ubiquitous tissue-specific knock down) and assays that can be performed to study the phenotypes resulting from mitochondrial disease. The high degree of conservation of the subunits of complex I in *Drosophila* allow us to closely model mitochondrial diseases.

In this study, a drawback of *Drosophila* was the difficulty in interpreting some specific behaviours as these were directly dependent on, or intertwined with, other behaviours such as locomotion and the search for food.

> **"We try to answer a question, sometimes with some ideas or expectations, and it is crucial to stay prepared for unexpected results."**

What has surprised you the most while conducting your research?

When we conduct an experiment, we start with a hypothesis. We try to answer a question, sometimes with some ideas or expectations, and it is crucial to stay prepared for unexpected results. Two points in this study were surprising and challenging. First, the abdominal shrinkage that we initially thought was due to accelerated aging, but soon understood to result from feeding difficulties when we provided them with a liquid solution of 5% sucrose with Bromophenol Blue. This phenotype, identified in dNDUFS4 KD flies, actually mimics the feeding problems experienced by patients with Leigh syndrome.

The second surprising point originates from the fact that patients with NDUFS4 mutation suffer from impairment of motor skills, among other symptoms. We therefore assessed the spontaneous locomotion abilities of the dNDUFS4 KD flies. It was counterintuitive that solid-fed (starving) dNDUFS4 KD flies walked significantly more than the liquid-fed (fed) dNDUFS4

Sarah Foriel's contact details: Khondrion BV, P.O. Box 9101, M357.00.132, Nijmegen 6500 HB, The Netherlands.
E-mail: foriel@khondrion.com

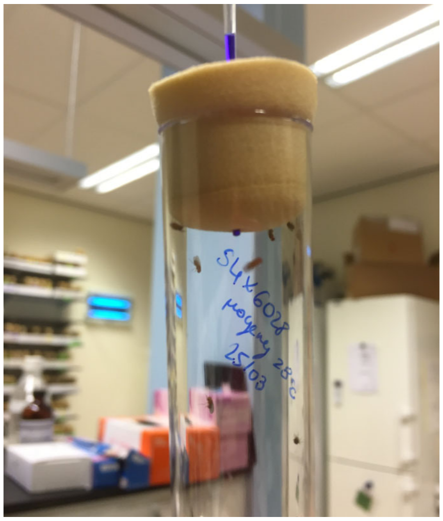

dNDUFS4 KD flies present a dramatic feeding defect that can be partially ameliorated by liquid feeding through capillaries.

KD flies that we expected to perform better. In fact, the outcome of this assay was influenced by both the feeding status and the health status of the flies. Starved flies probably walk more to forage for food despite their mitochondrial deficiencies, while the fed knockdown flies probably stayed more still to conserve energy.

Describe what you think is the most significant challenge impacting your research at this time and how will this be addressed over the next 10 years?

At this time, the main challenge lies in the extreme heterogeneity of clinical presentation in mitochondrial diseases; time of onset, genes/tissues/organs concerned, symptoms and poor diagnosis. This is where animal models can contribute to building knowledge and understanding.

The other challenge concerns the screening of new therapeutics, since no treatments are currently available for patients. A choice of readouts to evaluate potential therapeutics for screening is crucial and needs to meet the mode of action of these therapeutics.

What's next for you?

The next step for me is to find a postdoctoral position. I would love to continue in the field of mitochondrial disease using *Drosophila* as a model but let's see where the future takes me.

Reference

Foriel, S., Beyrath, J., Eidhof, I., Rodenburg, R. J., Schenck, A. and Smeitink, J. A. M. (2018). Feeding difficulties, a key feature of the *Drosophila* NDUFS4 mitochondrial disease model. *Dis. Model. Mech.* **11**: dmm032482, doi:10.1242/dmm.032482.
